# A fully automated platform for photoinitiated RAFT polymerization†

**DOI:** 10.1039/D2DD00100D

**Published:** 2023-01-05

**Authors:** Jules Lee, Prajakatta Mulay, Matthew J. Tamasi, Jonathan Yeow, Molly M. Stevens, Adam J. Gormley

**Affiliations:** a Department of Biomedical Engineering, Rutgers, The State University of New Jersey, Piscataway, NJ 08854, USA; b Department of Materials, Department of Bioengineering, and Institute of Biomedical Engineering, Imperial College London, London, SW7 2AZ, UK; c Department of Medical Biochemistry and Biophysics, Karolinska Institutet, SE-171 77 Stockholm, Sweden

## Abstract

Oxygen tolerant polymerizations including Photoinduced Electron/Energy Transfer-Reversible Addition–Fragmentation Chain-Transfer (PET-RAFT) polymerization allow for high-throughput synthesis of diverse polymer architectures on the benchtop in parallel. Recent developments have further increased throughput using liquid handling robotics to automate reagent handling and dispensing into well plates thus enabling the combinatorial synthesis of large polymer libraries. Although liquid handling robotics can enable automated polymer reagent dispensing in well plates, photoinitiation and reaction monitoring require automation to provide a platform that enables the reliable and robust synthesis of various polymer compositions in high-throughput where polymers with desired molecular weights and low dispersity are obtained. Here, we describe the development of a robotic platform to fully automate PETRAFT polymerizations and provide individual control of reactions performed in well plates. On our platform, reagents are automatically dispensed in well plates, photoinitiated in individual wells with a custom-designed lightbox until the polymerizations are complete, and monitored online in real-time by tracking fluorescence intensities on a fluorescence plate reader, with well plate transfers between instruments occurring *via* a robotic arm. We found that this platform enabled robust parallel polymer synthesis of both acrylate and acrylamide homopolymers and copolymers, with high monomer conversions and low dispersity. The successful polymerizations obtained on this platform make it an efficient tool for combinatorial polymer chemistry. In addition, with the inclusion of machine learning protocols to help navigate the polymer space towards specific properties of interest, this robotic platform can ultimately become a self-driving lab that can dispense, synthesize, and monitor large polymer libraries.

## 1 Introduction

Synthetic polymers are ubiquitous in modern industry with diverse applications in medicine such as pharmaceutical excipients, implants, and tissue engineering scaffolds.^1,2^ The versatility of polymeric materials stems from their tunability of a multitude of different chemical and physical properties, such as polymer molecular weight, hydrophobicity/hydrophilicity, crystallinity, charge, and mechanical properties. However, the discovery of precise polymer compositions that display desired structure–function relationships is challenging and time-consuming due to the exploration of an immense chemical landscape. Recently, the use of automation and robotics in the laboratory has enabled high-throughput screening of structure–function relationships and enabled faster and more efficient polymer discovery. For example, the pharmaceutical industry now utilizes high-throughput screening to explore properties of interest within a defined parameter space.^3–5^ Furthermore, robotics such as autosamplers and plate readers have found increasing use within laboratory settings to automate laborious and repetitive processes to increase throughput and minimize labor. Current trends in laboratory automation are moving towards more advanced forms of automation, where robotic platforms can perform entire experiments. In the case of polymer chemistry, robotics and automation can provide powerful tools for combinatorial chemistry, where large libraries of diverse polymers are synthesized in high throughput to keep up with the demand for new and innovative materials.^4,6,7^

Controlled/Living Radical Polymerization (CLRP) *via* Reversible Addition–Fragmentation Chain Transfer (RAFT) polymerization has been previously used to generate libraries of new and innovative polymers in parallel for biomedical applications.^8–15^ Recently, multiple research groups have developed impressive automated platforms specifically for high-throughput polymer synthesis. For example, Lin *et al*. reported a platform technology that allowed for the generation of a two-dimensional library of 100 diblock polyester and polycarbonate copolymers in under nine minutes using continuous-flow reactors.^16^ Additionally, Leibfarth and co-workers developed a flow system that accelerated the synthesis of sequence-defined copolymers by semiautomated iterative exponential growth.^17^ Chen and co-workers used a droplet flow system to generate 11 statistical copolymers within 11 minutes.^18^ However, the reaction monitoring must be performed manually, which slows down the overall development of novel polymers. Therefore, few groups have further improved these platform technologies by including automated characterization methods into the workflow. For example, Warren and Junkers demonstrated operator-independent online monitoring of RAFT polymerizations *via* flow chemistry using an inline benchtop NMR.^19,20^ Meanwhile, Lauterbach *et al*. reported the use of an inline UV-Vis spectrophotometer to monitor the flow polymerization online.^21^ Junkers recently incorporated inline size-exclusion chromatography (SEC) and NMR, which is a promising platform that may also enable high-throughput parallel synthesis, kinetic screenings, and robust characterization of polymers.^22^ While such automated platforms provide great utility for flow-based RAFT polymerization methods, there is an unmet need for the development of a platform that generates polymer libraries in a batch process with real-time monitoring of reaction conversions.

**Fig. 1:**
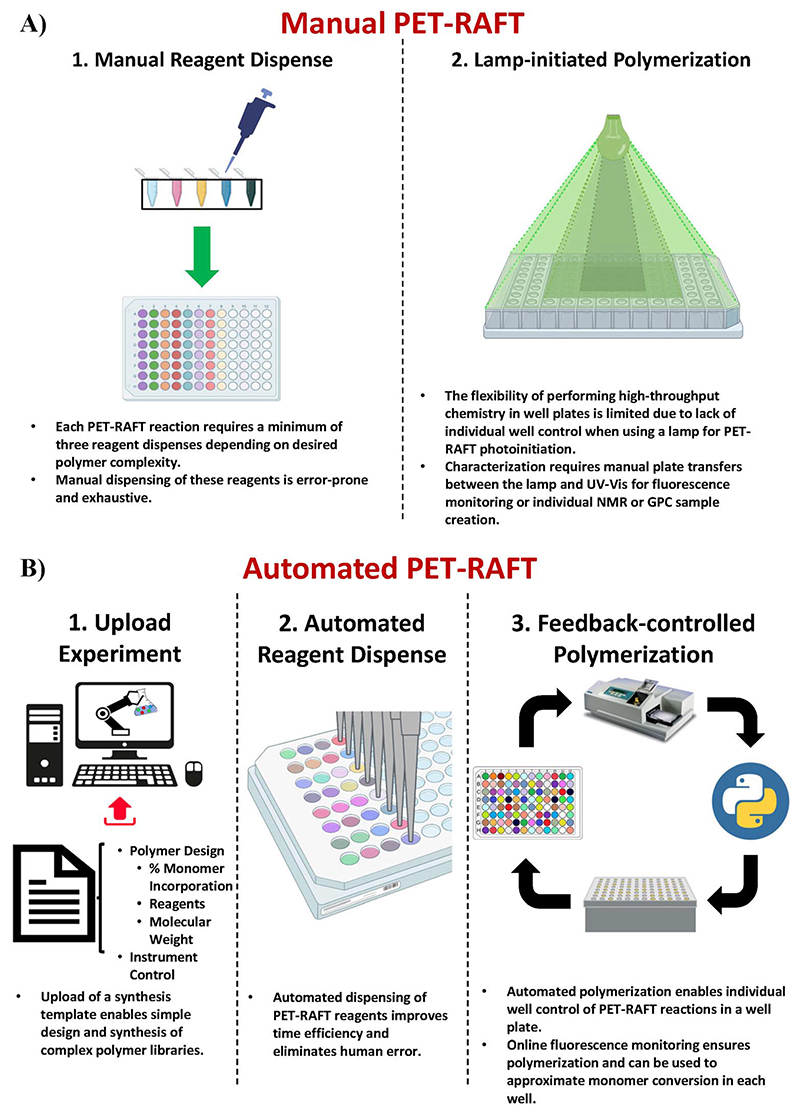
(A) Manual PET-RAFT polymerizations require (l) reagent dispensing into individual wells of a 96-well plate manually, which is prone to pipetting, positional, or volumetric errors, and (2) use of an overhead lamp to initiate PET-RAFT reactions that restricts control over individual reactions throughout the plate. (B) Automated PET-RAFT polymerization allows for (1) simple custom polymer design by uploading a synthesis template consisting of reaction parameters such as monomer content and degree of polymerization, (2) automated dispensing of reagents using liquid handling robotics to eliminate any human error associated with manual pipetting steps while reducing time and improving efficiency, and (3) automated reaction monitoring using online fluorescence tracking and automated spatial and temporal control over individual reactions using a lightbox with 96 multiplexed LEDs.

**Fig. 2:**
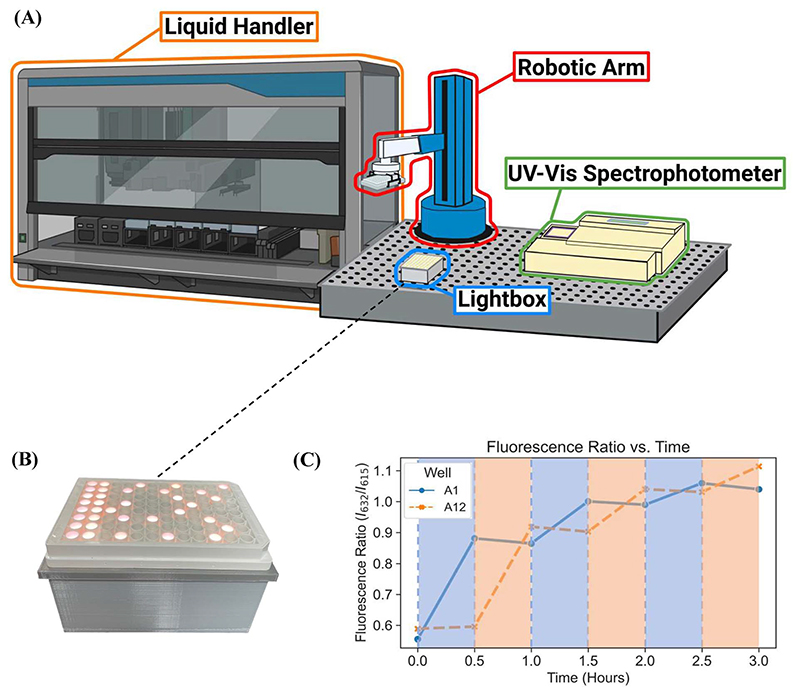
(A) The automated platform contains a liquid handling robot, our custom-designed lightbox for PET-RAFT initiation, a UV-vis spectro-photometer plate reader for online reaction monitoring, and a robotic arm to facilitate plate transfers between instruments. (B) 96-well plate containing PET-RAFT reaction mixtures placed on our lightbox. The fluorescence data of the reaction mixture at *t* = 0 is used to automatically control LED lighting profiles on the lightbox which resulted in wells only containing PET-RAFT reactions being lit for photoinitiation. (C) Fluorescence ratio *vs*. time plot of lightbox-initiated pDMA PET-RAFT polymerization in wells A1 and A12 of a single 96 well plate using warm-white LEDs. Shaded areas represent LED activation and polymer photoinitiation of their respective corresponding wells.

The discovery of oxygen-tolerant Photoinduced Electron/Energy Transfer-Reversible Addition–Fragmentation Chain Transfer (PET-RAFT) polymerization has enabled polymerizations to be performed in a well plate format on the benchtop.^23–26^ Boyer and co-workers used a photo-initiator that served a dual purpose of initiating the polymerization as well as aiding the singlet oxygen trapping for facilitating oxygen tolerance, whereas Gibson and co-workers achieved oxygen tolerance using triethanolamine as the degassing agent.^24,27–29^ This oxygen tolerance has further enabled the development of high-throughput polymer chemistry.^30^ However, creating polymer libraries in well plate formats requires manually adding the reagents in individual wells which is time-consuming and low-throughput (Fig. 1A-1). Our group recently demonstrated the utilization of liquid-handling robotics with PET-RAFT polymerizations that allowed for high-throughput synthesis of combinatorial polymer libraries in well plate format (Fig. 1B-1 and B-2).^4^ This liquid handling system reduces >80% of the time required for reagent dispensing and allows for a fully automated platform for combinatorial polymer chemistry. However, the use of high-throughput photochemistry in well plates requires additional challenges to be overcome to achieve a fully robotic platform.

The first challenge for end-to-end automated PET-RAFT polymerizations is providing automated control over the photoinitiation of individual reactions in each well. Previous experiments have demonstrated that the initiation of a PET-RAFT reaction is solely dependent on exposure to light, which provides control over polymerization initiation and termination.^31,32^ Conventional methods for PET-RAFT polymerization require that the well plate containing the reaction mixture be placed directly under an LED lamp or an array.^24,31,33,34^ However, these methods are limited in performing combinatorial PET-RAFT synthesis of various polymer compositions since they do not offer control over individual reactions in each well (Fig. 1A-2). For example, excess light exposure in one well may result in continuous RAFT activation in the absence of monomer leading to undesirable side reactions such as loss of RAFT end group functionality or even polymer coupling events that would skew the desired polymeric attributes such as the molecular weight and dispersity. A solution to this limitation is the design of a device with addressable LEDs in which the lighting of individual wells is controlled to provide spatial and temporal control of PET-RAFT reactions and enable combinatorial polymer chemistry in which each polymer attains the desired molecular weight and dispersity. 96-LED lightboxes are also used in biological research.^35,36^ For example, Amuza Inc provides a commercially available 96-LED matrix for opto-genetic applications, while Henkel's LOCTITE® LED flood system uses an array of UV or visible light LEDs for adhesive curing.^37^ However, these lightbox designs are limited in their applications for high-throughput PET-RAFT polymerization since there is (i) no control over individual LEDs, (ii) limited compatibility with automation, (iii) are expensive costing over $6000, and (iv) are not available in multiple wavelengths. Thus, a lightbox with individually addressable LEDs that can independently provide optimal light exposure for robust and reliable high-throughput combinatorial well plate PET-RAFT polymerizations is desired to enable a highly precise level of control over polymerization.

Significant potential also exists to further automate PET-RAFT polymerizations and improve control over the polymerization process by integrating techniques for *in situ*, continuous monitoring of the reaction progress. Yeow *et al*. have recently shown that using 5,10,15,20-tetraphenyl-21*H*,23*H*-porphine zinc (ZnTPP) as a photocatalyst for PET-RAFT reactions allows for online monitoring of the reaction by tracking changes in the photocatalyst fluorescence.^32^ They observed that the evolution of the ratio of the emission intensities at 632 and 615 nm (*I*_632_/*I*_615_) was highly correlated to the evolution of monomer conversion and thus allowing PET-RAFT polymerizations to be monitored in real-time.

**Fig. 3:**
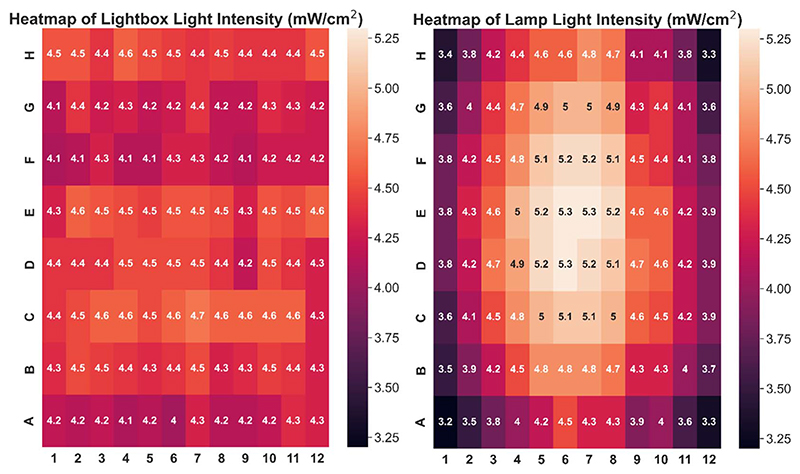
Heatmap of light intensities at different well positions using (left) lightbox with all LEDs on (variance = 2.3%) and (right) an overhead lamp (variance = 29.2%).

Here, we report a novel platform technology consisting of a custom-designed lightbox, a robotic arm, and a fluorescence plate reader (Fig. 1B-3) for automating PET-RAFT polymerizations in well plates to obtain a library of polymers in parallel. The custom-built lightbox is able to individually address LEDs that are automatically controlled throughout the polymerization using Python. It can be easily fabricated at a low cost of approximately $100 and allows for customizations for specific purposes depending on the user. In addition, these polymerizations are tracked *via* online fluorescence monitoring on the spectrophotometer without manual intervention. Before initiating the polymerization with the lightbox, fluorescence intensities are obtained, and the fluorescence data is then imported to Python where fluorescence ratios are calculated and plotted.

The data is then sent to the lightbox to control the individual LEDs. The robotic arm then performs a plate transfer from the fluorescence plate reader to the lightbox to initiate PET-RAFT polymerizations, then transfers back to the fluorescence plate reader for another fluorescence read after 30 minutes of photopolymerization. This process is repeated until Python calculates that the slopes between the two most recent consecutive fluorescence data time points are below a set threshold for each reaction in the well plate and then deems the polymerizations to be complete. The LEDs are then automatically turned off for the completed polymerizations by Python. Thus, this robotic platform allows for fully automated online fluorescence tracking of PET-RAFT polymerizations and enables a method to provide an extremely high level of spatial and temporal control of individual PET-RAFT reactions in a well plate format. Finally, building upon our previous work, we also report the facile integration of Hamilton Microlab STARlet liquid handling robotics^4^ with our robotic platform to enable high-throughput polymerizations of acrylate homopolymers and copolymers synthesized in parallel.

## 2 Experimental

### 2.1 Lightbox prototyping and characterization

For lightbox prototyping and design, 74HC595 8-bit shift registers (Catalog #46105) and UDN2981A current source drivers (Catalog #1762681) were purchased from Jameco Electronics (CA, USA). 3000 K warm white light-emitting diodes (LEDs) were purchased from Digi-Key (Catalog #2138-JK2835BWT-W-U30EB0000-N0000001CT-ND – Cut Tape (CT)) (MN, USA). A custom-designed printed circuit board (PCB) was created using Eagle software on the Fusion 360 platform and was fabricated by OSH Park. The lightbox was created according to the schematic shown in (Fig. S1†) using an LM2309 DC-DC step-down converter connected to an external power source.^38^ The DC–DC step-down converter was supplied 24 V and was stepped down to 8.5 V to supply power to the UDN2981A. In addition, 3D CAD models for the lightbox shell were generated on SketchUp V5.0.2 and 3D printed in-house using a Creality Ender 5 3D printer to contain all components and allow 96-well plates to be seated on top of the LED array. Finally, an Arduino sketch written in the Arduino IDE was uploaded to the lightbox Arduino and included the functions that enabled communication with the master Python script. A binary data array calculated in Python which was based on fluorescence reads performed on the spectrophotometer was used to control the LED multiplexing. All lightbox files and resources are listed in a GitHub repository (**https://doi.org/10.5281/zenodo.7379060**). Light intensity measurements at 560 nm were performed using a Newport optical power meter (Model 1917-R). Measurements for the lightbox were obtained from the top, and measurements for the lamp were obtained at four inches from the bulb. These distances were used to best represent the intensity of light experienced by each reaction well using different light sources.

### 2.2 Automated fluorescence tracking

Python 3.10.0 software was used to handle data analysis and control each instrument. The first step consisted of taking an initial fluorescence measurement using Molecular Devices SpectraMax® M2 fluorescence plate reader of a 96-well plate containing PET-RAFT polymerization reaction mixtures in individual wells. The excitation wavelength (*E*_x_) was set to 410 nm, and the fluorescence intensities at the emission wavelengths (*E*_m_) of 615 and 632 nm were obtained. Fluorescence intensity measurements were obtained and exported as an XML file.

The saved XML file was then imported to Python to be analyzed for lightbox control. Empty wells that showed negligible fluorescence values were replaced with a “0” value, and the fluorescence ratios for wells containing the reaction mixture were calculated using eqn (1),

(1) $$I_{t_{0}}=\frac{I_{632}}{I_{615}}$$

where *I*_615_ is the fluorescence intensity at 615 nm, *I*_632_ is the fluorescence intensity at 632 nm, and *I*_*t*_0__ is the fluorescence intensity ratio and *t* = reaction time, wherein this case *t* = 0.

This 8 × 12 matrix for 96 wells was saved internally to later display a list of fluorescence ratios for wells containing PET-RAFT reactions. A copy of the fluorescence data matrix was further manipulated in Python to ultimately control LED lighting. The fluorescence data matrix was converted into a binary logic array, *A_ij_*, where non-zero fluorescence values were replaced with a “1” value, while the “0” values remained. *A_ij_* was then multiplied by matrix *B_j_* shown in eqn (2),

(2) $$B_{j}=2^{j}$$

where *j* ranges from 1 to 12. Matrix A and B were multiplied to result in matrix *C_ij_* as shown in eqn (3),

(3) $$C_{i}=\sum_{j}A_{ij}B_{j}$$

where *i* ranges from 1 to 8 and *j* ranges from 1 to 12. This resulted in a data array representative of the 96-well plate, where the *i*th element of C encoded which LEDs to turn on in each row. Matrix C was then sent to the Arduino controlling the lightbox, allowing for controlled lighting of individual wells (Fig. S2†).

After the initial fluorescence read, a Hudson Robotics PlateCrane EX robotic arm was used to perform plate transfers between the fluorescence plate reader and the lightbox. PETRAFT polymerizations were allowed to proceed for 30 minutes and were then transferred from the lightbox back to the fluorescence plate reader by the robotic arm. The fluorescence read, plate transfer, and lighting calculations previously described were repeated, and the slope of the line joining the two consecutive fluorescence ratios was determined using eqn (4),

(4) $$\text{slope}\;=\frac{I_{t_{2}}-I_{t_{1}}}{30}$$

where $I_{t_{2}}=\frac{I_{632}}{I_{615}}$ at *t* = *t*_2_, and $I_{t_{1}}=\frac{I_{632}}{I_{615}}$ at *t* = *t*_1_.

If the calculated slope at the two most recent time points of a reaction well was lower than a set threshold, as indicated by the plateauing of the slope, the polymerization reaction in the well was deemed complete and the corresponding LED was turned off. The automation of the polymerization reactions continued until the obtained slope for each reaction well was below the set threshold.

### 2.3 PET-RAFT polymerizations

#### 2.3.1 Materials

4-Acryloylmorpholine (NAM), *N,N*-dimethylacrylamide (DMA), 2-hydroxyethyl acrylate (HEA), methyl acrylate (MA), ethyl acrylate (EA), (2-methoxyethyl) acrylate (MEA), *N*-isopropylacrylamide (NIPAM) monomers, RAFT chain transfer agent (CTA): 2-(dodecylthiocarbonothioylthio)pro-pionic acid, and dimethyl sulfoxide (DMSO) were purchased from Sigma-Aldrich (MO, USA). ZnTPP was purchased from VWR (PA, USA).

#### 2.3.2 PET-RAFT polymerization of multiple acrylate/acrylamide homopolymers

Stock solutions of NAM (2 M), DMA (2 M), BA (2 M), HEA (2 M), MA (2 M), EA (2 M), MEA (2 M), CTA (50 mM), and ZnTPP (2 mM) were prepared in DMSO. For each homopolymer, stock solutions of individual monomer, CTA, and ZnTPP were dispensed manually into wells of a white-walled 96-well polystyrene plate to prevent off-well activation (Thermo Fisher Scientific, Catalog #265302) to the desired volume using a [*M*_0_] : [CTA] : [ZnTPP] = 200 : 1 : 0.02 to obtain a target degree of polymerization (DP) of 200. A clear plate sealing film was applied to the top of the plate to prevent solvent evaporation and contamination. The well plate was then placed in the fluorescence plate reader for *t* = 0 fluorescence reading. The reaction mixtures were photoinitiated using either our custom-built lightbox or a green 560 nm lamp. The polymerizations were monitored using fluorescence tracking. For light-box photoinitiation, the PlateCrane EX robotic arm initiated the plate transfers between the fluorescence plate reader and the lightbox to automatically monitor and photo-initiate PET-RAFT polymerization reactions, respectively. Unless otherwise stated, the polymerizations were monitored using our robotic platform and online fluorescence tracking for automated control of LEDs of the lightbox (multiplexing) to individually control each reaction where the experiment was performed until Python determined all wells to be complete. 10 μL aliquots of each homopolymer were obtained every 30 minutes to calculate conversion through ^1^H-NMR spectroscopy. 20 μL aliquots of each homopolymer were also obtained to determine the molecular weights and dispersity using SEC.

#### 2.3.3 High-throughput automated PET-RAFT polymerization of homopolymers and copolymers

A template (Table S1†) containing the composition of the polymers to be synthesized was uploaded to Python, which controlled the Hamilton Microlab STARlet liquid handling robot to dispense reagents into a white-walled 96-well plate. Monomer, CTA, and ZnTPP stock solutions were prepared as described above and loaded into the Hamilton Microlab STARlet liquid handling robot.^4^ Reagents were dispensed into the well plate by the liquid handling robot. Then, the PlateCrane transferred the plate containing the reaction mixtures from the Hamilton to the fluorescence plate reader to start the online fluorescence monitoring. The automated platform initiated the polymerizations *via* the lightbox and monitored the reactions with fluorescence tracking until all polymerizations were determined to be completed. 10 μL aliquots for ^1^H-NMR spectroscopy were obtained manually for all polymerization reactions at *t* = 0 and after all polymerizations were complete. Aliquots were also obtained for SEC analysis.

### 2.4 Size-exclusion chromatography (SEC)

SEC was performed using an Agilent 1260 Series instrument to determine polymer molecular weight (*M*_w_ and *M*_n_) and polydispersity (*Đ*). Samples were run with dimethylformamide (DMF) (VWR Industrial, Catalog #BDG83634.400) and lithium bromide (LiBr) (Sigma-Aldrich, Catalog #213225-100 G) as eluent through a Phenomenex 5.0 μm guard column (50 × 7.5 mm^2^) preceded by two Phenomenex Phenogel columns (10^4^ and 10^3^ Å). Calibration was performed using Agilent polymethylmethacrylate (PMMA) standards, and samples were prepared at a 1 : 50 polymer reaction mixture/eluent volume ratio in DMF and filtered using 0.45 μm PTFE filters.

### 2.5 Nuclear magnetic resonance (NMR) spectroscopy

Proton (^1^H)-NMR spectroscopy was performed on a Bruker Avance Neo 500 MHz Liquid State NMR spectrometer to monitor monomer conversions. Samples were prepared at a 1 : 50 polymer reaction mixture/solvent volume ratio with 2 mM mesitylene (Fisher Scientific, Catalog #AAA11323AE) as an internal standard in DMSO-*d*_6_ solvent. Each sample was run with eight scans and a relaxation delay of one sec. NMR spectroscopy data was processed using Mestrenova 14.3.0 Lite software.

## 3 Results and discussion

### 3.1 Lightbox characterization

The custom-built lightbox designed specifically for high-throughput PET-RAFT polymerizations used multiple integrated circuits, an Arduino, a custom-designed PCB, and a voltage regulator connected to an external power source (Fig. S1†). The lightbox was installed onto an optical table and integrated with the automated platform by securing communication between Python and the lightbox Arduino, and a PlateCrane pick-up/drop-off point on top of the lightbox for transfers between the lightbox and fluorescence plate reader that monitored the progress of the reaction was also set (Fig. 2A). To test whether PET-RAFT reaction locations in the well plate could directly influence LED lighting on the lightbox, a single-step automated fluorescence tracking trial was performed using a dummy plate containing PET-RAFT reactions in the first two columns, and an additional twenty random wells were used. Lightbox multiplexing proved to be successful, as only the wells containing PET-RAFT reactions had their corresponding LEDs activated (Fig. 2B). Optical power measurements at 560 nm taken for each individual LED on the lightbox showed that intensities ranged from 4 to 4.7 mW cm^−2^ with a variance of only 2.3% compared to measurements taken at corresponding locations from the lamp which displayed radially-distributed light intensities ranging from 3.2 to 5.3 mW cm^−2^ with a variance of 29.2% (Fig. 3). These differences in spectral wavelength profiles, light intensities, and light homogeneities could possibly affect overall reaction kinetics, the warm white LEDs employed on the lightbox were still hypothesized to enable effective PET-RAFT initiation. The use of warm white LEDs was required for the lightbox due to the high amount of surface heat radiated onto the 96-well plate when using yellow-green or lime-green LEDs, which caused solvent evaporation onto the protective film and affected fluorescence measurements. Thus, it was hypothesized that the automated platform with our custom lightbox and automated fluorescence tracking could allow for fine control over PET-RAFT polymerization reaction in each well in a 96-well plate.

To validate the hypothesis that excess photoinitiation in a PET-RAFT polymerization could lead to over polymerization, two replicates of pDMA (DP 200) and pMEA (DP 200) were polymerized for three hours and 24 hours in the center wells of a polystyrene 96 well-plate. Comparison of SEC traces demonstrated that prolonged polymerization times led to the presence of distinct high molecular weight shoulders for both monomers (Fig. S3†). We hypothesized that the prolonged irradiation led to dimerization of the polymer chains due to continual activation of the RAFT end group. These side reactions are undesirable not only for the increase in polymer dispersity but also for the loss of end chain fidelity (livingness) which would severely limit additional chain extension of the polymer or the efficiency of post-modification reactions. They also show the formation of higher molecular weight populations indicated by the bimodal trace of pDMA and a shoulder in pMEA irradiated for 24 hours. The formation of these higher molecular weight products caused by the dimerization of polymer chains due to excess photoinitiation is undesirable as they negatively affect the dispersity of the polymer. In addition, these side products may affect the end chain fidelity and livingness of the reaction and hamper additional chain extension of the polymer in case of synthesis of block copolymers. Over-polymerization may deter performing combinatorial chemistries of different monomers with different reactivities throughout a single plate. Thus, a lightbox with individually addressable LEDs that controls the radiation times specifically tailored to each PET-RAFT polymerization in a well plate could ensure that high monomer conversions are reached while preventing the negative effects of over polymerization.

To further investigate the applicability of our custom-built lightbox for PET-RAFT polymerizations, two replicates of pDMA (DP 200) were synthesized in wells A1 and A12 of a 96-well plate, and their respective LEDs were periodically turned on and off every 30 minutes by the automated platform where the lighting of wells A12 and A1 alternated after every time point. Fluorescence tracking was performed at every timepoint to monitor the reaction progress of each well (Fig. 2C). The fluorescence ratio for well A1 increased in the first 30 minutes when its corresponding LED was on, however, the fluorescence ratio remained constant, and the monomer conversion ceased for the next 30 minutes when its LED was off. The fluorescence ratio increased again after the corresponding LED was turned on suggesting that the livingness of the polymerization and the photoactivity of ZnTPP were conserved. The response of the fluorescence ratios to the status of the LED was consistent throughout the experiment. Concurrently, a similar response was obtained for the polymerization in well A12. These results indicated that the inherent activation and inactivation character of PET-RAFT mediated by light exposure could be tightly regulated at the individual well level in well plates by the implementation of individually addressable LEDs. In contrast to previous work with PET-RAFT polymerization whereby a single light source was used to control the polymerization process of the entire well plate, our lightbox enabled additional dimensions of PET-RAFT control at the spatial and temporal levels in single wells of a 96-well plate.^32^

### 3.2 PET-RAFT polymerization of acrylate/acrylamide homopolymers

**Fig. 4:**
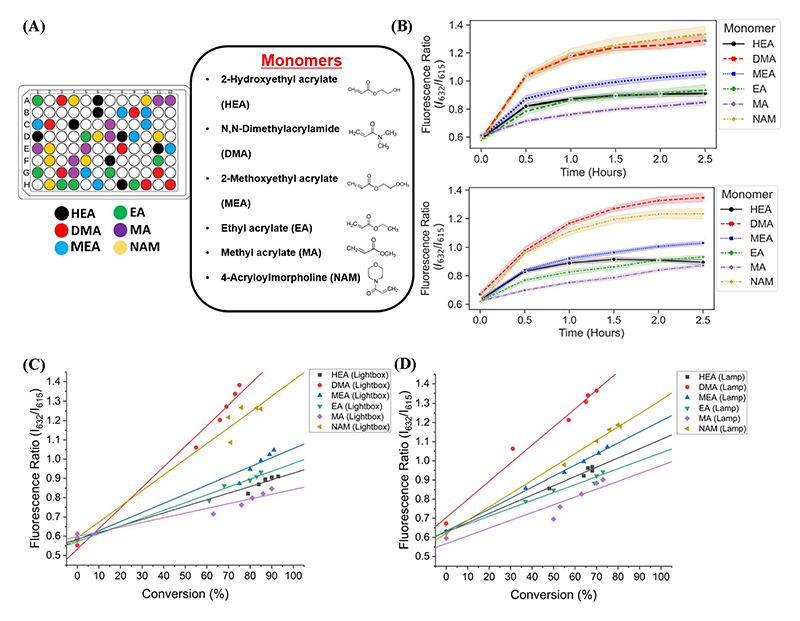
(A) A 96-well plate containing six different homopolymer reactions, each with eight replicates for a total of 56 used wells. Each well contained monomer, ZnTPP photocatalyst, CTA, and DMSO. (B) Fluorescence ratio vs. time plots of DMA, HEA, MA, MEA, and NAM polymerized on the lightbox (top) and lamp (bottom). Graphed lines represent the mean fluorescence ratio for all replicates, and shaded areas represent a 95% confidence interval. Correlations of fluorescence ratios and monomer conversions calculated by ^1^H-NMR spectroscopy of all homopolymers polymerized using (C) the lightbox with constant LED lighting and (D) the lamp.

We next sought to validate the use of our custom-designed lightbox to synthesize multiple homopolymer species and compare the performance to the lamp photoinitiated polymerization. We performed parallel PET-RAFT polymerization reactions where eight replicates of pHEA, pDMA, pMEA, pEA, pMA, and pNAM homopolymer reactions were dispensed randomly across a 96-well plate manually (Fig. 4A) and were polymerized on our automated platform. A second identical plate was created as well to undergo polymerization under a 560 nm LED lamp as a control. A data array was manually sent to the lightbox to produce a lighting pattern that corresponded to PET-RAFT reaction locations to simulate individual lighting of only wells containing polymerization mixtures, and our automated platform performed online fluorescence tracking for 2.5 hours. The lightbox lighting pattern was kept constant throughout the reaction to get a baseline of each monomer's response to photoinitiation under the lightbox.

The trend of the evolution of fluorescence ratios at different time points demonstrated by the lightbox and the lamp show similarity across all monomers except for pDMA, which displayed lower fluorescence ratio magnitudes on the lightbox than on the lamp (Fig. 4B). However, higher monomer conversions (calculated through ^1^H-NMR spectroscopy) were obtained with lightbox than lamp at all time points. All polymers synthesized on the lightbox demonstrated more than 90% final conversion except for pEA and pDMA, which reached a final conversion of 85% and 75%, respectively, and all polymers synthesized on the lamp showed final conversions ranging between 68% and 80%. The higher conversions observed using the lightbox may have been the result of the larger spectral profile of the lightbox compared to the lamp leading to an increased activation of ZnTPP when using warm-white LEDs as the photoinitiation source.^39,40^ Shanmugam *et al*. have reported that multiple color wavelengths can activate ZnTPP at different rates, where yellow, green, and orange light cause fast polymerization while colors such as red and blue result in relatively slower polymerizations.^40^ The broad absorbance of ZnTPP may therefore have caused increased ZnTPP activation and faster polymer reaction kinetics on the lightbox compared to the lamp. Interestingly, all homopolymers displayed linear correlations of the fluorescence ratio with monomer conversion consistent with the findings of Yeow *et al*. (Fig. 4C and D).^32^ The calculation of adjusted *R*^2^ linear correlation coefficients of all homopolymers indicated a high correlation between fluorescence ratios and conversions for all homopolymers regardless of photoinitiation source (Table S2†). pMA synthesized on the lightbox and lamp displayed correlation coefficients of 0.899 and 0.832, respectively, while all other homopolymers displayed correlation coefficients >0.95. Analysis of the correlation curves between homopolymers synthesized on the lightbox and those synthesized on the lamp show that although there was some variation in the fluorescence ratios and their associated conversions for each homopolymer, the lightbox was still able to synthesize PET-RAFT polymers similar to that of the lamp. The observation of high monomer conversions and strong linear correlations between fluorescence ratio and conversion for polymers synthesized on the lightbox provided strong evidence that our custom-designed lightbox could enable robust PET-RAFT reactions in parallel. In addition, we hypothesized that the use of automated LED multiplexing could allow for control of individual PET-RAFT reactions by using the slopes between fluorescence ratios calculated between consecutive time points to monitor reaction progress instead of the fluorescence ratio values as an indication of reaction termination.

**Fig. 5:**
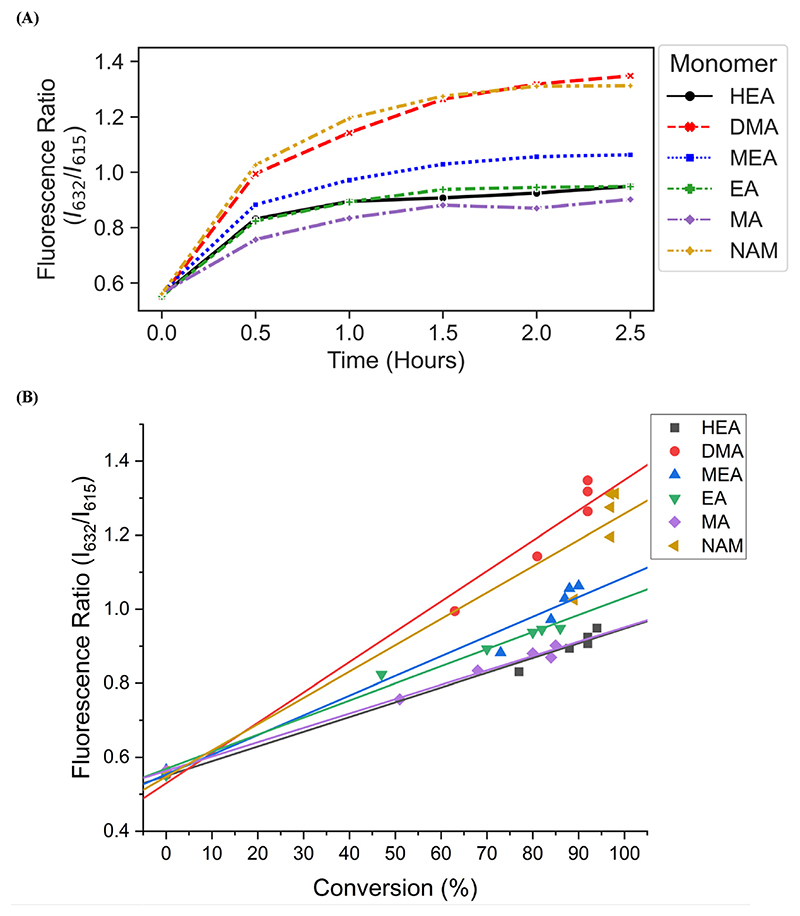
(A) Fluorescence ratio vs. time plots of pHEA, pDMA, pMEA, pEA, pMA, and pNAM homopolymers synthesized on the lightbox with LED multiplexing. (B) Correlation of polymer fluorescence ratio and monomer conversions calculated using ^1^H-NMR spectroscopy. Homopolymers were synthesized on the lightbox with feedback-controlled LED multiplexing.

We then investigated the utility of our automated platform and the impact of multiplexed lighting controlled by slopes of fluorescence ratios obtained between consecutive time points in each well on polymer attributes. A slope threshold of 0.002 was set in Python to ensure that all polymers synthesized received maximum light exposure and achieved high monomer conversions. Parallel PET-RAFT polymerization reactions were performed where reagents for pHEA, pDMA, pMEA, pEA, pMA, and pNAM were dispensed in the first column of a 96-well plate manually and were polymerized on our automated platform with feedback-controlled lighting. The slopes for fluorescence ratios *vs*. time plots of all acrylate monomers polymerized with the lightbox plateaued at *t* = 2 h time points, and thus their corresponding LEDs were switched off automatically, while NAM and DMA were deemed to be completely polymerized at *t* = 2.5 h by our platform (Fig. 5A). This difference in plateauing at different fluorescence ratio values may have been caused by the differences in ZnTPP (photocatalyst) incorporation into the different polymer backbones, as suggested by Yeow *et al*.^32^

The conversions of these homopolymerizations were determined using ^1^H-NMR spectroscopy. The conversion kinetics of the homopolymers that underwent LED multiplexed lighting were similar to those of their respective homopolymers synthesized using constant lightbox radiation (Fig. S4†). Final conversions obtained at 2.5 h for all polymers again showed high final conversions of above 85%, demonstrating that the use of multiplexed lighting provided automatic control of each polymerization while high final conversions were achieved. Linear correlations between the fluorescence ratios and the observed conversion at each time point calculated through ^1^H-NMR spectroscopy of homopolymers synthesized using feedback-controlled LED lighting again showed strong correlations, as *R*^2^ values for all polymers were >0.95 except for pNAM which displayed a lower *R*^2^ value of 0.89 (Fig. 5B, Table S3†). Since NAM demonstrated more than 85% conversion within the first time point of 30 minutes, we tracked NAM conversion on the lightbox with increased time resolution of 5 minutes (Fig. S5†) for optimizing the linear correlation fit. Similar fitting equations were obtained with both the time resolutions, that is *y* = 0.00625*x* + 0.57797 with *R*^2^ of 0.96 for a time resolution of 5 minutes as compared to *y* = 0.0071*x* + 0.54747 with *R*^2^ of 0.95 obtained with 30 minute time resolution, thus representing the true fit. In addition, an increase in the fluorescence ratio was observed at constant NAM conversion beyond 90 minutes leading to a poor fit. This increase may have been caused by additional fluorescence changes induced by the non-linear incorporation of ZnTPP into the polymer backbone at very high NAM conversions. Since the slope threshold of 0.002 for automated light cutoffs may have been too low for NAM polymerization, it over-exposed the NAM polymerization at high conversion that induced a non-linear incorporation of ZnTPP in the polymer backbone. To optimize the slope threshold for NAM polymerization, its conversion was tracked at a resolution of 15 minutes for 2.5 h (Fig. S6†). More than 95% conversion was obtained within 90 minutes where the slope of 0.00284 was still above the set threshold of 0.002 that continued the reaction further and thus increasing the fluorescence ratio. Therefore, for highly reactive species such as NAM, setting a higher threshold of 0.003 is suggested. This indicated that determining fluorescence ratios to provide tailored lighting times to each polymer reaction can provide correlations to the actual monomeric conversions when performing PET-RAFT polymerizations using either the lightbox or the lamp as the light source, but direct correlations between the fluorescence ratios and experimental conversions are dependent on the specific monomer used. In addition, these results indicate that the use of slope calculations between fluorescence ratios provides a method that enables each PET-RAFT reaction within the separate wells of a well plate to receive the optimal amount of light exposure to ensure full conversion and potentially minimize the effects of over-polymerization. Although there may be differences in final fluorescence ratio values, this system takes advantage of the fluorescence ratio plateauing behavior conserved throughout all PET-RAFT reactions to provide flexible and robust synthesis of different PET-RAFT polymers in parallel. Finally, we anticipate that the use of this fluorescence monitoring method will also be of particular utility in quality control processes to determine the progression of each PET-RAFT reaction.

To confirm polymerization was controlled according to a PET-RAFT mechanism, polymers were analyzed with SEC (Fig. S7, Table S4†). Comparisons between the SEC traces of homopolymers synthesized on the lamp *versus* the lightbox show that polymers synthesized on the lightbox showed similar molecular weights and molecular weight distributions thereby demonstrating our platform's ability to provide control over individual polymerization reactions. It should be noted that the molecular weights obtained for HEA homopolymers were higher than the theoretical molecular weight which was attributed to the difference in hydrodynamic diameter between pHEA and the PMMA standards. Homopolymers synthesized on the lightbox also demonstrated similar polymer dispersities compared to those obtained with the lamp. Overall, our custom-built lightbox demonstrated success in performing PET-RAFT polymerizations comparable to those synthesized using a conventional lamp-based illumination source and provided the advantage of automated individual well control for parallel polymer synthesis to ensure intermediate to high conversion while providing a mechanism for the prevention of over polymerization.

### 3.3 Fully automated PET-RAFT synthesis of multiple polymer compositions

**Fig. 6:**
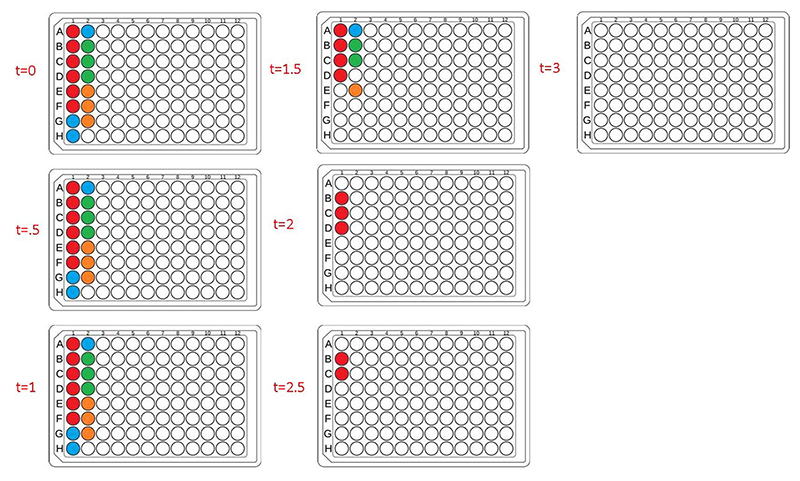
Lightbox lighting patterns of each 30 minute timepoint for a plate containing homopolymers and copolymers synthesized in parallel. Colored wells indicate that the corresponding LED for that well is on, where red = homopolymers, blue = copolymers with 50 : 50 monomer ratio, green = copolymers with 33 : 33 : 33 monomer ratios, and orange = copolymers with 25 : 25 : 25 : 25 monomer ratios. White wells indicate that the corresponding LED for that well is off, thus indicating feedback control of multiplexed lighting and photoinitiation.

Next, to fully automate more diverse PET-RAFT polymerizations, we employed the use of a Hamilton liquid handling robot for automatic dispensing of reagents to synthesize multiple homopolymers and copolymers in parallel. The reaction mixtures for homopolymers and copolymers underwent feedback-controlled multiplexed light exposure until Python determined that each individual reaction was complete, following which the corresponding LED was automatically turned off. The multiplexed light exposure time was automatically determined using the slope of the fluorescence ratios *vs*. time plots at regular intervals of time (30 minutes) and the lights were switched off when the polymerizations were deemed complete by the platform (Fig. 6).

**Fig. 7:**
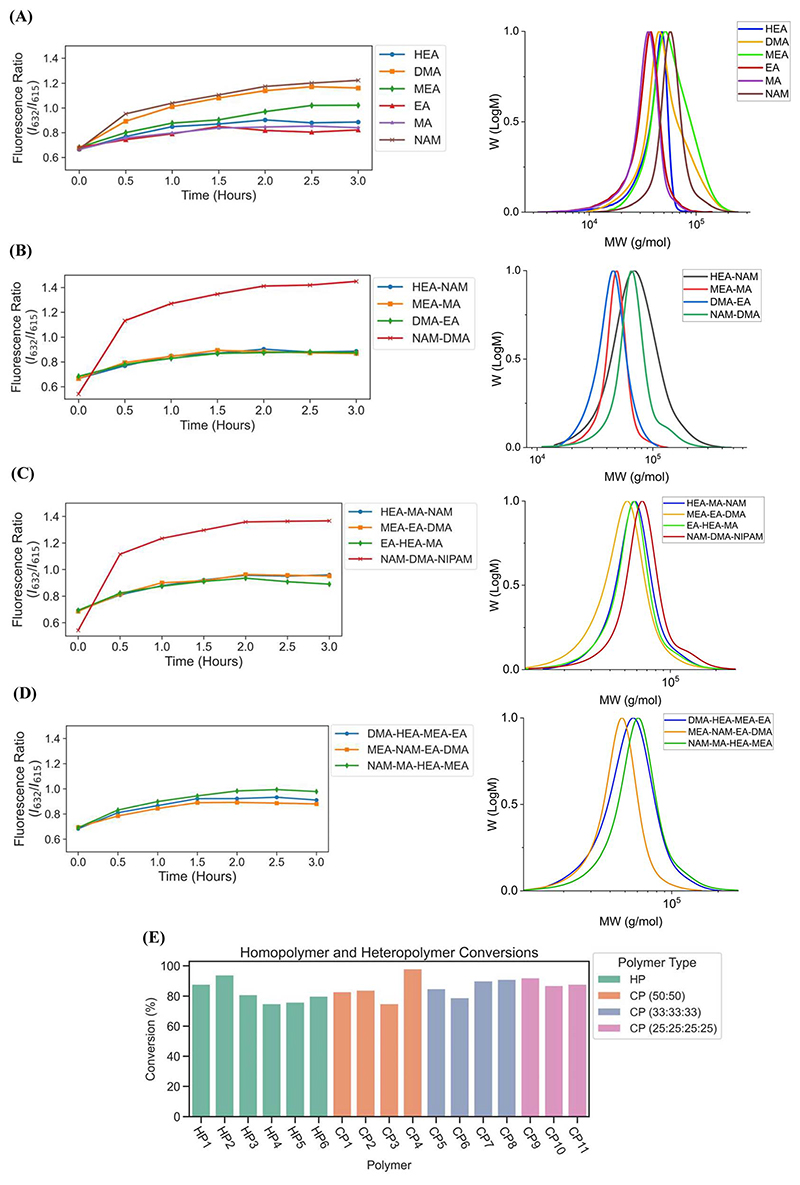
Fluorescence ratio vs. time graphs of (A) pHEA, pDMA, pMEA, pEA, pMA, and pNAM homopolymers, (B) copolymers with 50 : 50 monomer ratios, (C) copolymers with 33 : 33 : 33 monomer ratios, and (D) copolymers with 25 : 25 : 25 : 25 monomer ratios synthesized on the automated platform with feedback-controlled LED multiplexing. (E) Conversions of all monomers synthesized on the automated platform and calculated using ^1^H-NMR spectroscopy. Homopolymers (HP) and copolymers (CP) were completed after three hours of polymerization.

Copolymers synthesized using our automated robotic platform demonstrated fluorescence ratios similar to those of the homopolymers, where the evolution of fluorescence ratio for each polymer with time displayed a logarithmic trend (Fig. 7A–D). The slopes for fluorescence ratios *vs*. time plots of all the copolymers containing acrylate monomers plateau between *t* = 1 h and *t* = 2 h, and therefore the reaction was deemed complete, and their LEDs were turned off automatically. All copolymers containing exclusively acrylamides were completed after *t* = 3 h. CP1, CP2, CP7, CP10, and CP11 copolymers with HEA-*co*-NAM, MEA-*co*-MA, EA-*co*-HEA-*co*-MA, DMA-*co*-HEA-*co*-MEA-*co*-EA, and MEA-*co*-NAM-*co*-EA-*co*-DMA compositions were synthesized by 1.5 h; and CP3, CP5, CP6, and CP9 with DMA-*co*-EA, HEA-*co*-MA-*co*-NAM, MEA-*co*-EA-*co*-DMA, and NAM-*co*-MA-*co*-HEA-*co*-MEA compositions were completely polymerized by 2 h. For homopolymer reactions, EA and MA were deemed to be completely polymerized at 1.5 h, HEA at 2 h, DMA at 2.5 h, and NAM and MEA at 3 h. In addition, copolymers containing only acrylamide monomers: NAM-*co*-DMA and NAM-*co*-DMA-*co*-NIPAM, displayed the same behavior as their homopolymer counterparts in which they reached high fluorescence ratios even after full conversion, yet this behavior was prevented with the incorporation of an acrylate monomer into the copolymer composition. Despite the variation in fluorescence ratios, all homopolymers and copolymers showed intermediate to high conversions, where total conversions of homopolymers ranged from 75 to 94%, copolymers with two monomer compositions ranged from 75 to 98%, copolymers with three monomer compositions ranged from 79 to 90%, and copolymers with four monomer compositions ranged from 88 to 92% (Fig. 7E) as calculated using ^1^H-NMR spectroscopy. Intermediate to high conversions were obtained for each monomer independent of desired polymer composition, which showed the versatility of using fluorescence tracking for synthesizing acrylate/ acrylamide homopolymers and copolymers. SEC traces further demonstrated desired molecular weights (Fig. 7A–D, Table S5†), however, the molecular weights obtained for copolymers containing HEA were higher than the theoretical molecular weight due to the swelling of HEA in solution. It was expected that the *Đ* of the copolymers to be slightly higher than the homopolymers due to the different reactivities between different monomer families in solution, but good control over the polymerization using our automated platform was demonstrated with relatively low dispersities throughout. Synthesis of both homopolymers and copolymers with control of each well using our automated platform resulted in polymer synthesis with high final conversions and good agreement between the theoretical and observed molecular weights while obtaining low dispersities. In addition, the use of slope calculations to determine the completion of reactions proved to be flexible enough to provide robust polymer synthesis regardless of monomer composition and provided strong evidence that our automated platform can be a powerful tool for high-throughput combinatorial chemistry in well plates.

## 4 Conclusion

In this work, a custom-designed lightbox with multiplexed LED lighting was developed to provide automatic individual control over PET-RAFT reactions performed in a 96-well plate. In addition, lightbox lighting was automatically controlled by using Python scripts to develop a robotic platform in which fluorescence ratios calculated in each well were used to provide feedback regarding reaction completion after each subsequent fluorescence read to switch individual LEDs on or off. Testing of the lightbox and its utility in driving the synthesis of multiple different acrylate and acrylamide homopolymers in parallel displayed tailored lighting to individual wells based on fluorescence ratio feedback for each homopolymer species and resulted in effective polymerizations comparable to those performed using an overhead lamp. Furthermore, we confirmed the use of slope calculations between sequential fluorescence ratio calculations as a method to directly control PET-RAFT initiation and provide robust polymer synthesis. We also scaled up the platform's synthesis capabilities by including the use of a Hamilton liquid handler to dispense a series of different homopolymer and copolymer compositions for polymerization using our automated platform. The robust synthesis of different polymer compositions with high monomer conversions and low dispersity demonstrated the efficacy of using our automated workflow for a multitude of polymer designs while supporting the potential use of this robotic platform to develop combinatorial libraries of complex polymer designs with relative ease.

This automated platform could be further challenged to synthesize larger polymer libraries that consist of methacrylate and methacrylamide monomers, as well as different polymer architectures such as block copolymers and star copolymers. Methacrylate and methacrylamide monomers demonstrated slow changes in fluorescence intensity ratio lower than the set threshold of 0.002 between 30 minute time points which caused early termination of reaction by Python (not reported), and therefore, optimization of time points is required for each family of monomers. This caveat as well as the fact that a slope threshold of 0.002 seems to be too low for acrylamide homopolymers underlines how our automated platform could be vastly improved by assigning a slope threshold to each individual reaction based on which monomer species is being used. The versatility of this automated platform can be further improved by the use of specifying lighting patterns for each timepoint using the experimental template by entering the binary data array to provide control over lighting times in each well. The creation of this automated platform also opens the door to the inclusion of different automated workflows and assays. For example, the addition of another lightbox with UV LEDs would allow for automated side chain functionalization of polymer backbones synthesized on our platform and allow for increased exploration into polymer structure–function relationships.^41^

Our automated platform can be further expanded by integration of additional instruments, as well as the addition of automated data analysis workflows. For example, analytical techniques essential for polymer characterization such as SEC could be optimized by automating processes for sample preparation and trace analysis using the liquid handler and Python, respectively. Furthermore, the use of high-throughput SEC columns allowing run times of four minutes per sample at the sacrifice of resolution would significantly improve analytical capacity. In addition, the inclusion of more experimental steps in Python could allow for automated enzyme kinetics assays, and the addition of a dynamic light scattering plate reader would allow for high-throughput hydrodynamic radius screening of different polymer compositions. Finally, further development of this platform with the inclusion of machine learning protocols would enable decisive and effective navigation of the highly multidimensional space that different polymer compositions and architectures provide. With the large amounts of data that automation can provide in the field of polymer chemistry, multiple groups have already shown how machine learning can take full advantage of these large datasets. Warren *et al*. were able to feed data generated by an online NMR and inline GPC into a closed-loop machine learning algorithm to optimize polymerization reaction conditions and directly influence polymer characteristics such as dispersity and conversion.^42^ Similarly, Leibfarth *et al*. demonstrated the use of active machine learning combined with automated polymer synthesis to identify effective ^19^F MRI copolymer agents.^43^ Hartman *et al*. utilized an artificial neural network to predict metallocene-catalyzed olefin polymerizations.^44^ In our own work, the inclusion of active learning with our automated platform informs polymer synthesis and characterization until the desired polymer structure–function behavior is found, such as polymers that self-assemble into single-chain polymer nanoparticles or polymers that can form polymer-protein hybrids.^6,7,45^ This self-driving laboratory in which a design-build-test-learn paradigm is employed to systematically discover new polymers with specific activities will allow nonexperts to create highly complex polymer designs reliably and efficiently.

## Supplementary Material

Supporting information

## Data Availability

All data discussed in the manuscript is included in the supplemental materials. Files containing resources for the lightbox, including Python and Arduino code, 3D print files, circuit schematics, and PCB manufacturing files are available in a GitHub repository at https://doi.org/10.5281/zenodo.7379060.
