# Supplementary material for: A fully automated platform for photoinitiated RAFT polymerization
Source: Digit Discov. Author manuscript; Available in PMC 2024 Dec 8. (PMC7616994; doi:10.1039/D2DD00100D)
Supplement: Supporting information [file EMS193200-supplement-Supporting_information.pdf]

Electronic Supplementary Material (ESI) for Digital Discovery.
This journal is © The Royal Society of Chemistry 2023

Supporting Information

A Fully Automated Platform for Photoinitiated RAFT Polymerization

Jules Lee,[a] Prajakatta Mulay,[a] Matthew J. Tamasi,[a] Jonathan Yeow,[b] Molly M. Stevens,[b, c] Adam J. Gormley*, [a]

[a] Department of Biomedical Engineering, Rutgers, The State University of New Jersey, Piscataway, NJ 08854, USA

[b] Department of Materials, Department of Bioengineering, and Institute of Biomedical Engineering, Imperial College London, London, SW7 2AZ, U.K.

[c] Department of Medical Biochemistry and Biophysics, Karolinska Institutet, SE-171 77 Stockholm, Sweden

*Correspondence to:

Adam J. Gormley: adam.gormley@rutgers.edu

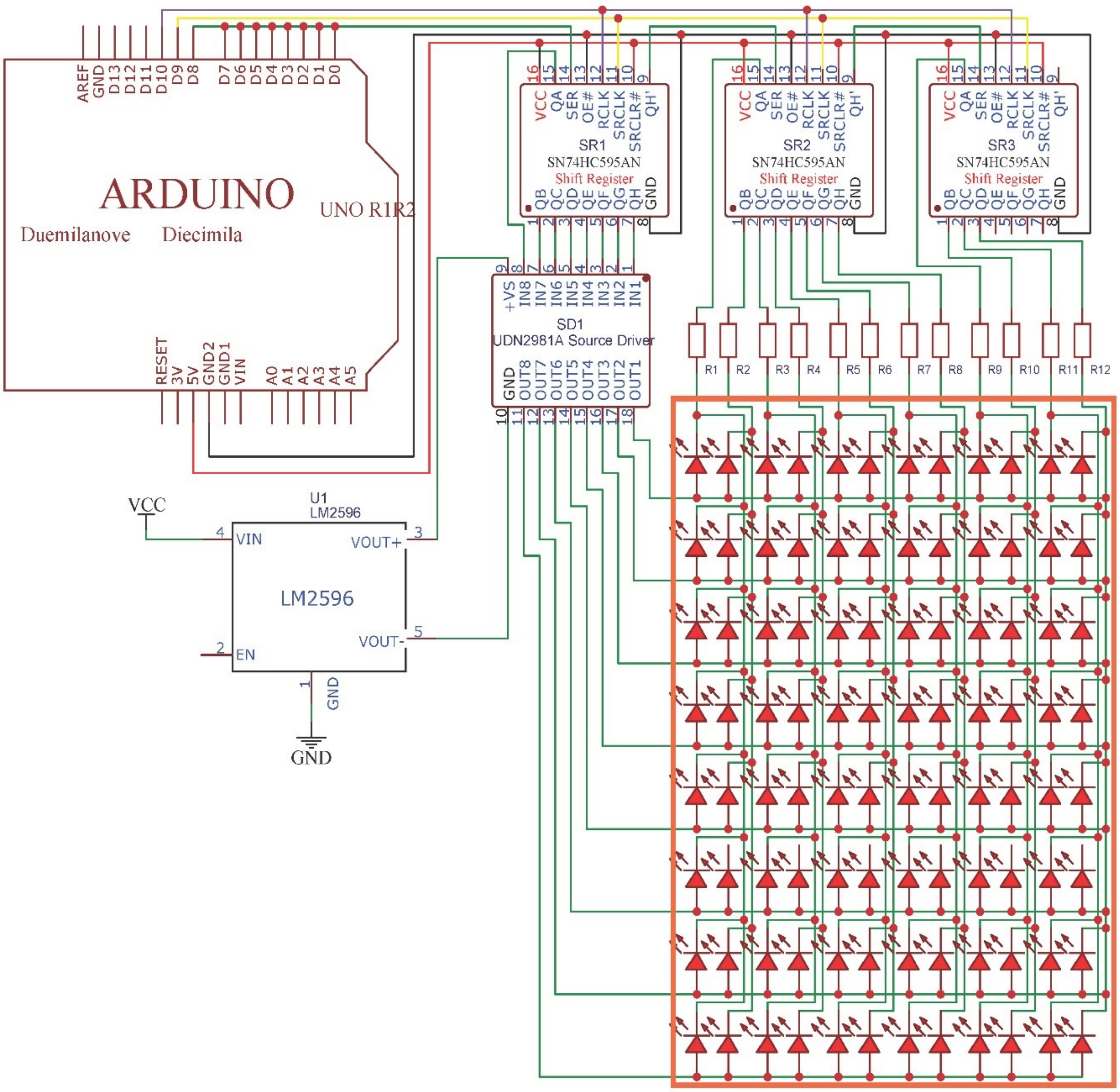

Figure S1. Circuit schematic of lightbox with multiplexed LEDs consisting of an Arduino, three shift registers, a current source drive, voltage regulator module, and 96 LEDs in a dot-matrix configuration.

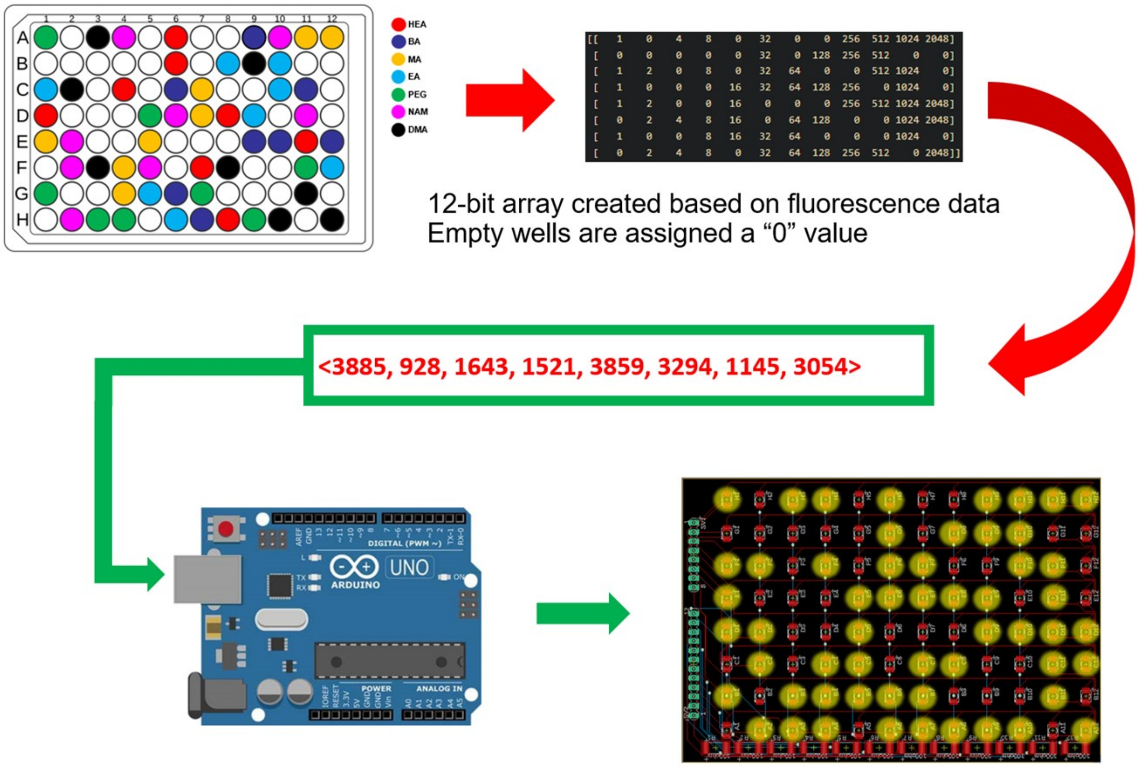

Figure S2. Workflow for automated fluorescence tracking and LED lighting. After a fluorescence read is done by the UV-Vis, the ratios are converted into a binary data matrix, where wells are represented by a binary number. The sum of all numbers in a specific row is sent to the Arduino, where each element in the array represents the sum of its respective row. The Arduino can then drive the shift registers to control which wells are lit.

Table S1. Synthesis template to be executed by the Hamilton liquid handling robot for homopolymers and heteropolymers.

	Polymer ID	Degree of Polymerization	Volume	CTA	Mon 1	% Mon 1	Mon 2	% Mon 2	Mon 3	% Mon3	Mon 4	% Mon4
**0**	1	400	200	6	HEA	100		0		0		0
**1**	2	400	200	6	NAM	100		0		0		0
**2**	3	400	200	6	MEA	100		0		0		0
**3**	4	400	200	6	DMA	100		0		0		0
**4**	5	400	200	6	EA	100		0		0		0
**5**	6	400	200	6	MA	100		0		0		0
**6**	7	400	200	6	HEA	50	NAM	50		0		0
**7**	8	400	200	6	MEA	50	MA	50		0		0
**8**	9	400	200	6	DMA	50	EA	50		0		0
**9**	10	400	200	6	NAM	50	DMA	50		0		0
**10**	11	400	200	6	HEA	33	MA	33	NAM	33		0
**11**	12	400	200	6	MEA	33	EA	33	DMA	33		0
**12**	13	400	200	6	EA	33	HEA	33	MA	33		0
**13**	14	400	200	6	NAM	33	DMA	33	NIPAM	33		0
**14**	15	400	200	6	NAM	25	MA	25	HEA	25	MEA	25
**15**	16	400	200	6	DMA	25	HEA	25	MEA	25	EA	25
**16**	17	400	200	6	MEA	25	NAM	25	EA	25	DMA	25

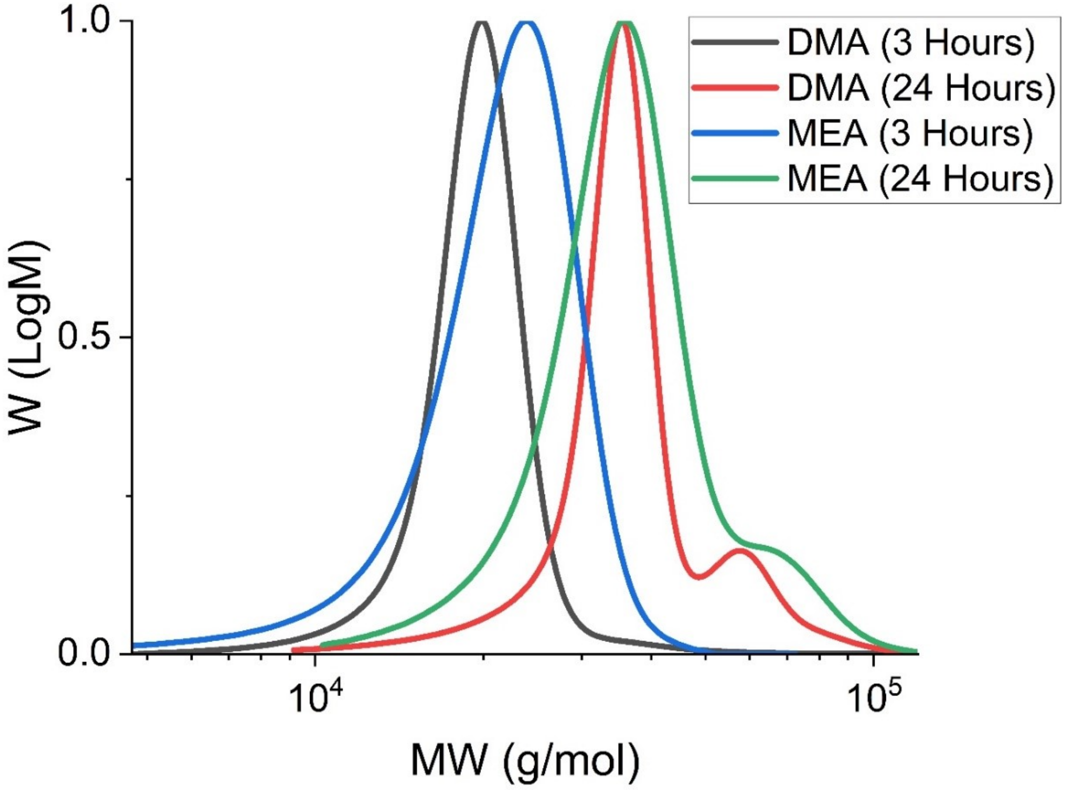

Figure S3. SEC traces of DMA and MEA polymerized using the lamp-initiated PET-RAFT for 3 hours and 24 hours. Samples polymerized for 24 hours displayed significantly increased changes in molecular weight due to chain coupling compared to samples polymerized for only three hours.

Table S2. Correlation coefficients for homopolymers synthesized using the lightbox without LED multiplexing and the lamp.

Polymer	Lightbox R^2	Lamp R^2
HEA	0.976	0.990
DMA	0.979	0.977
MEA	0.947	0.997
EA	0.975	0.977
MA	0.899	0.832
NAM	0.951	0.995

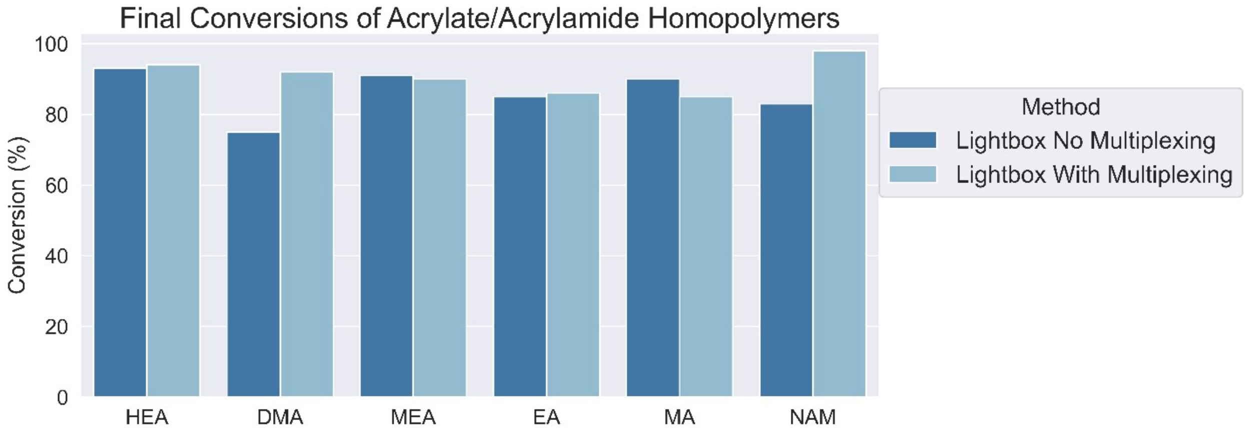

Figure S4. Final acrylate and acrylamide homopolymer conversions calculated through ^{1}H-NMR after polymerization using the lightbox. Homopolymers were initiated with either constant LED lighting for 2.5 hours (Lightbox No Multiplexing) or initiated with individual LED control based on fluorescence ratio slopes calculated using the automated platform (Lightbox With Multiplexing).

Table S3. Linear correlation coefficients of the correlation between the homopolymer fluorescence ratios and conversions calculated using ^{1}H-NMR spectroscopy. Homopolymers were synthesized on the automated platform with multiplexed LED lighting to control each PET-RAFT reaction.

Polymer	R^2
HEA	0.983
DMA	0.968
MEA	0.953
EA	0.979
MA	0.991
NAM	0.890

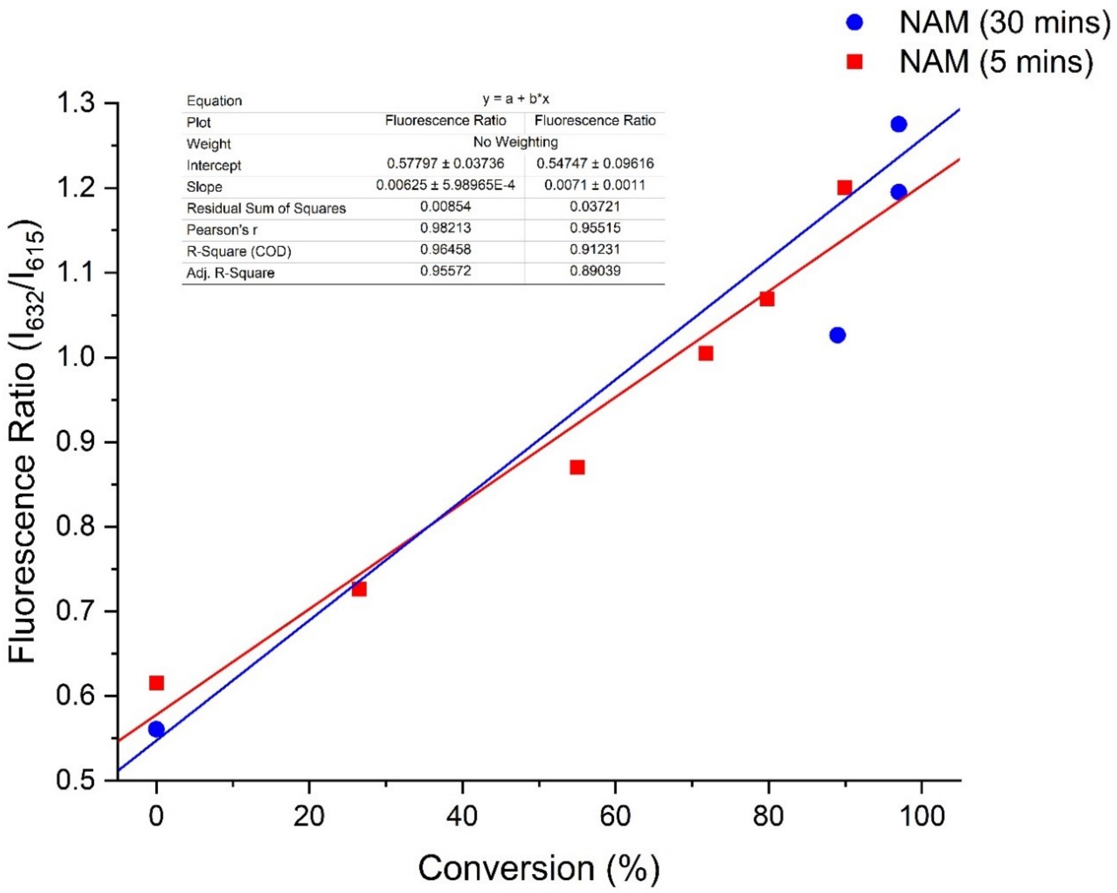

Figure S5. Correlation between fluorescence ratio and conversion of pNAM polymerized for 30 minutes using 5-minute timepoints (red squares) and for 2.5 hours using 30-minute timepoints (blue circles) including their respective linear fit curves. Both polymer reactions were performed on our automated platform using the lightbox.

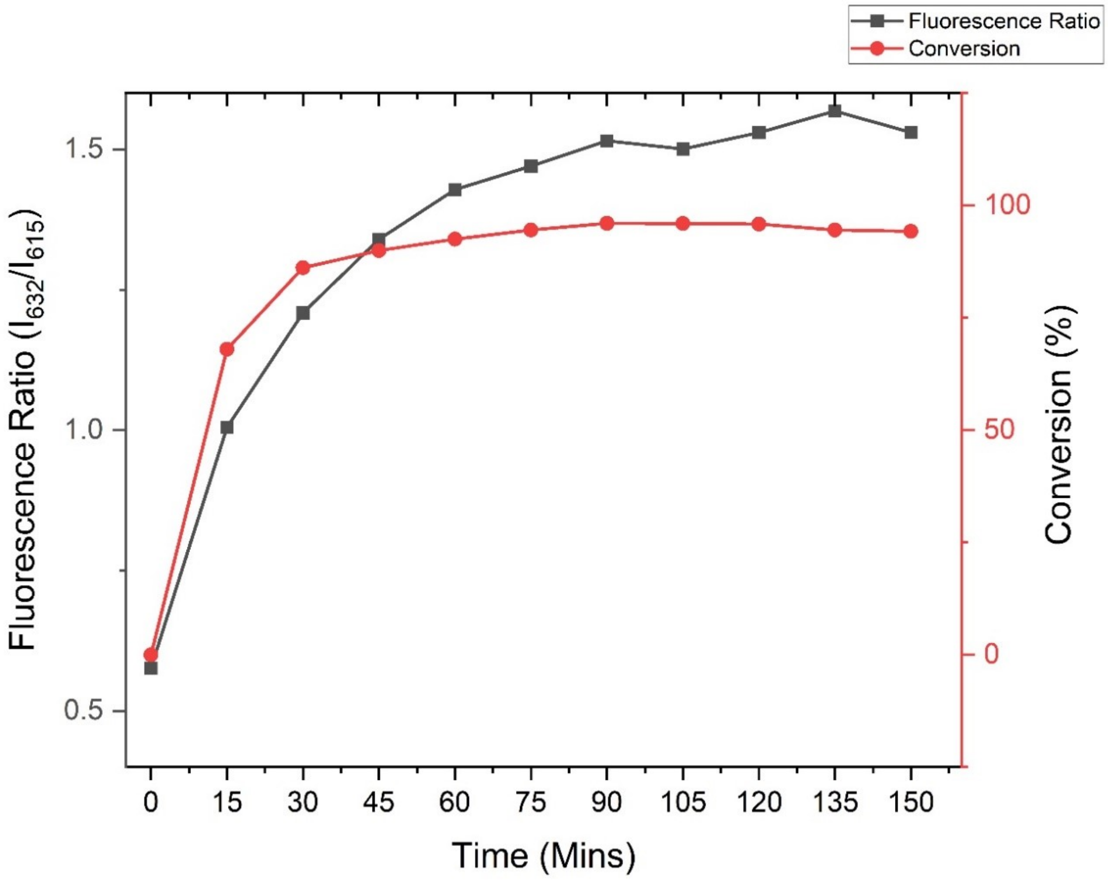

Figure S6. Plots of fluorescence ratio vs. time and conversion vs. time of pNAM polymerized for 2.5 hours using 15-minute timepoints with LED multiplexing.

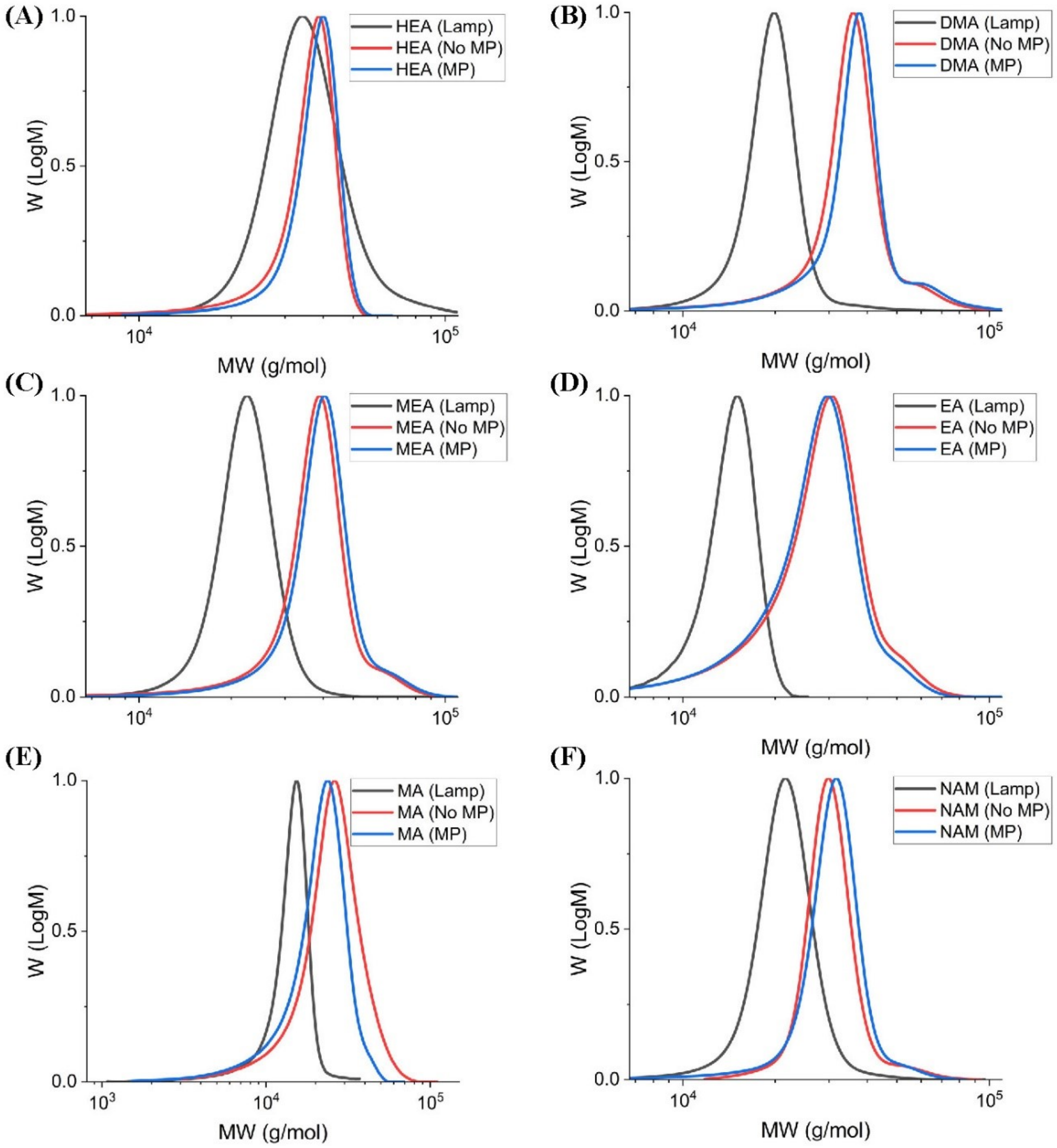

Figure S7. SEC traces of (A) DMA (B) EA (C) HEA (D) MA (E) MEA and (F) NAM polymerized on the lamp (black), the lightbox without LED multiplexing (red), and the lightbox with LED multiplexing (blue).

Table S4. SEC results of all homopolymers of HEA, DMA, MEA, EA, MA, and NAM synthesized on the lightbox and lamp

**Polymer**	$\boldsymbol{Mn_{Theor}}$ **(g/mol)**	**Lightbox (No MP)** $\boldsymbol{Mn_{GPC}}$ **(g/mol)**	**Lightbox (MP)** $\boldsymbol{Mn_{GPC}}$ **(g/mol)**	**Lamp** $\boldsymbol{Mn_{GPC}}$ **(g/mol)**	**Lightbox (No MP)** ***Đ***	**Lightbox (MP)** ***Đ***	**Lamp** ***Đ***
HEA	23,200	32,942	35,473	33,386	1.08	1.06	1.10
DMA	19,800	21,373	18,741	19,876	1.10	1.19	1.19
MEA	26,000	34,078	36,213	21,363	1.12	1.11	1.06
EA	20,000	22,388	22,003	13,404	1.28	1.25	1.06
MA	17,200	21,212	17,632	12,311	1.25	1.26	1.16
NAM	28,200	29,595	28,939	20,482	1.05	1.09	1.06

Table S5. List of homopolymers and heteropolymers synthesized using our fully automated platform listing monomers used in each composition **(Polymer)**, polymer ID **(Polymer ID)**, polymer composition (Composition), theoretical molecular weight **(Mn_{Theor})**, observed molecular weight **(Mn_{SEC})**, dispersity **(*Đ*)**, and monomer conversion **(α)** calculated using ^{1}H-NMR spectroscopy

Polymer	Polymer ID	Composition (Monomer Ratio)	Mn_{Theor} (g/mol)	Mn_{SEC} (g/mol)	*Đ*	α(%)
HEA	HP1	Homopolymer	46,400	37,000	1.09	88
NAM	HP2	Homopolymer	56,400	54,779	1.15	94
MEA	HP3	Homopolymer	52,000	52,042	1.26	81
DMA	HP4	Homopolymer	39,600	51,908	1.16	75
EA	HP5	Homopolymer	40,000	55,830	1.18	76
MA	HP6	Homopolymer	34,400	28,787	1.13	80
HEA-NAM	CP1	Heteropolymer (50:50)	51,400	61,225	1.29	83
MEA-MA	CP2	Heteropolymer (50:50)	43,200	42,698	1.12	84
DMA-EA	CP3	Heteropolymer (50:50)	39,800	37,854	1.16	75
DMA-NAM	CP4	Heteropolymer (50:50)	48,000	61,239	1.17	98
HEA-MA-NAM	CP5	Heteropolymer (33:33:33)	45,722	53,095	1.17	85
MEA-EA-DMA	CP6	Heteropolymer (33:33:33)	43,856	42,526	1.23	79
EA-HEA-MA	CP7	Heteropolymer (33:33:33)	40,256	51,908	1.16	90
NAM-DMA-NIPAM	CP8	Heteropolymer (33:33:33)	46,949	59,349	1.16	91
NAM-MA-HEA-MEA	CP9	Heteropolymer (25:25:25:25)	47,300	55,830	1.18	92
DMA-HEA-MEA-EA	CP10	Heteropolymer (25:25:25:25)	44,500	48,362	1.19	87
MEA-NAM-EA-DMA	CP11	Heteropolymer (25:25:25:25)	47,000	39,976	1.16	88
